# Editorial: New functional separation and analysis technologies utilizing human Fc receptors

**DOI:** 10.3389/fimmu.2024.1434438

**Published:** 2024-06-06

**Authors:** Tilman Schlothauer, Dietmar Reusch, Manfred Wuhrer

**Affiliations:** ^1^ Pharma Research and Early Development, Roche Innovation Center Munich, Penzberg, Germany; ^2^ Pharma Technical Development Penzberg, Roche Diagnostics GmbH, Penzberg, Germany; ^3^ Center for Proteomics and Metabolomics, Leiden University Medical Center, Leiden, Netherlands

**Keywords:** Fc receptors, analysis technologies, molecular innate immunity, effector cells, adaptive and innate immunity

Understanding the impact of post-translational modifications requires advanced separation technologies to analyze functional antibody subpopulations.

This Research Topic aims to review current technologies for separating antibody mixtures, emphasizing biologically relevant species critical for clinical development. It also explore new insights into heterogeneous antibody preparations, focusing on tools like engineered cells and advanced analytics to assess Fc diversity and functionality, with CD16, CD32, CD64, and neonatal Fc receptors being of particular interest.

The studies in this Research Topic have provided significant insights into the interactions between Immunoglobulin G (IgG) proteoforms, Fc gamma receptors (FcγRs), and their implications for therapeutic antibody development and disease treatment. Here, we summarize key findings from five research articles that explore these interactions in various contexts.

In “Function-structure approach reveals novel insights on the interplay of Immunoglobulin G 1 proteoforms and Fc gamma receptor IIa allotypes” by Lippold et al., the authors investigate the relationship between IgG1 proteoforms and FcγRIIa allotype binding. The two FcγRIIa allotypes differ in a single amino acid at position 131. This difference affects immunological responses and the clinical outcomes of monoclonal antibodies (mAbs). The study introduces a function-structure-based approach using FcγRIIa affinity chromatography-mass spectrometry (AC-MS) assays to assess individual IgG1 proteoforms, revealing FcγRIIa allotype-specific differences in proteoform-resolved IgG1 binding. The paper goes beyond the mere resolution of IgG glycoforms and studies the effect of thermal stress-induced deamidation on IIa receptor interaction, pointing towards the importance of a hydrogen bond in causing allotype-specific effects of heat stress-induced deamidation on IIa affinity. This method refines structure-function relationships of IgG1 glycoforms and streamlines the assessment of critical quality attributes (CQAs) for therapeutic mAbs [fimmu-14–1260446 (2).pdf].

Fox et al., in “Enhancing the therapeutic activity of hyperimmune IgG against chikungunya virus using FcγRIIIa affinity chromatography,” explore the potential of improving the therapeutic activity of CHIKV-specific antibodies through enhanced FcγR engagement. They use FcγRIIIa-based affinity chromatography to enrich CHIKV-immune IgG for glycoforms with enhanced Fc receptor binding. The enriched IgG showed increased clearance of infectious virus and viral RNA, as well as enhanced neutrophil phagocytic activity, demonstrating the utility of FcγRIIIa-affinity chromatography in optimizing antibody therapeutics against CHIKV [fimmu-14–1153108.pdf].

In “Negative interference with antibody-dependent cellular cytotoxicity mediated by rituximab from its interactions with human serum proteins,” Yanaka et al. address the issue of therapeutic antibodies like rituximab, which can be negatively impacted by interactions with serum proteins. These interactions can interfere with the antibody’s ability to mediate antibody-dependent cellular cytotoxicity (ADCC), a key mechanism for eliminating cancer cells. The study highlights the importance of understanding and mitigating such interactions to maintain the therapeutic efficacy of antibodies.

Szittner et al. present “Cellular surface plasmon resonance-based detection of anti-HPA-1a antibody glycosylation in fetal and neonatal alloimmune thrombocytopenia.” This study uses cellular surface plasmon resonance to detect glycosylation patterns of anti-HPA-1a antibodies, which are involved in fetal and neonatal alloimmune thrombocytopenia (FNAIT). Understanding and monitoring the glycosylation of these antibodies could lead to better diagnostic and therapeutic strategies for FNAIT. It is expected that in many more conditions assessment of the glycosylation, other structural features, proteoforms and functionality of specific antibodies will be of great clinical benefit, and the cellular SPR platform may, due to its versatility and multiplexing capacity, prove very valuable for developing such assays suitable for clinical translation.

Lastly, Gstöttner et al., in “Benchmarking glycoform-resolved affinity separation – mass spectrometry assays for studying FcγRIIIa binding,” focus on the development of assays to study the binding of IgG glycoforms to FcγRIIIa. These assays are crucial for understanding how different glycoforms affect the efficacy of therapeutic antibodies, as FcγRIIIa binding is associated with ADCC activity. The paper compares affinity liquid chromatography (LC) approaches with affinity capillary electrophoresis (CE) approaches, both using online mass spectrometric detection for resolving glycoform heterogeneity and determining IgG glycoform-specific migration positions. Notably, while the separation principles are very different with elution being achieved using a pH stress in the case of LC, versus complex-induced mobility shifts in CE, the resulting insights into differential IgG1 proteoform specificity of FcγRIIIa are remarkably comparable. The study benchmarks these methods to provide a toolbox of reliable approaches for analyzing the interactions between IgG glycoforms and FcγRIIIa. Overall, the study makes an important step towards positioning *in vitro* FcγRIIIa binding assays as a surrogate for cell-based ADCC assays in the development of IgG1 therapeutics.

Together, these studies underscore the complexity of IgG-FcγR interactions and their relevance to antibody-based therapies. They highlight the need for advanced analytical techniques to dissect these interactions and optimize antibody design for improved clinical outcomes.

## Author contributions

TS: Writing – original draft, Writing – review & editing. DR: Writing – original draft, Writing – review & editing. MW: Writing – original draft, Writing – review & editing.

